# Building strong grant writers in academic medicine: outcomes of early-career faculty enrolled in the University of California San Diego Health Sciences Grant Writing Course

**DOI:** 10.1093/acamed/wvaf031

**Published:** 2026-01-12

**Authors:** Andrea Z LaCroix, Danielle Fettes, Yelda Serin, Mariko Poupard, Virginia Hazen, Deborah Wingard, JoAnn Trejo

**Affiliations:** Herbert Wertheim School of Public Health and Human Longevity Science, University of California San Diego, La Jolla, CA, United States; UC San Diego Health Sciences Office of Faculty Affairs, University of California San Diego, La Jolla, CA, United States; UC San Diego Health Sciences Office of Faculty Affairs, University of California San Diego, La Jolla, CA, United States; Department of Psychiatry, School of Medicine, University of California San Diego, La Jolla, CA, United States; UC San Diego Health Sciences Office of Faculty Affairs, University of California San Diego, La Jolla, CA, United States; UC San Diego Health Sciences Office of Faculty Affairs, University of California San Diego, La Jolla, CA, United States; UC San Diego Health Sciences Office of Faculty Affairs, University of California San Diego, La Jolla, CA, United States; Herbert Wertheim School of Public Health and Human Longevity Science, University of California San Diego, La Jolla, CA, United States; UC San Diego Health Sciences Office of Faculty Affairs, University of California San Diego, La Jolla, CA, United States; UC San Diego Health Sciences Office of Faculty Affairs, University of California San Diego, La Jolla, CA, United States; Department of Pharmacology, School of Medicine, University of California San Diego, La Jolla, CA, United States

**Keywords:** basic science education, faculty development, mentoring/coaching, program evaluation, workforce training and development

## Abstract

The success of early-career faculty at R1 research-intensive institutions depends on institutions’ ability to establish an independent, grant-funded research program in a highly competitive funding environment in which only 2,174 of 11,463 National Institutes of Health (NIH) applications (19%) submitted by early-stage investigators were funded in 2023. This report summarizes outcomes of early-career faculty enrolled in the University of California San Diego Health Sciences Grant Writing Course (GWC), which provided a structured, step-by-step, multicomponent experience focused on preparing a competitive grant proposal. The program evaluation includes effects on grant submission and funding rates and grant-writing self-efficacy after 2 years of follow-up. Eighty-five early-career faculty members were enrolled in the GWC from 2017 to 2021, including 48 (56%) MD and MD-PhD physicians, 37 (44%) PhD faculty, 45 (53%) women, and 15 (18%) self-identifying as being from underrepresented racial or ethnic backgrounds. Data from 82 participants (98%) at 12 or 24 months were used for grant outcomes, and 75 participants (88%) with 12- and 24-month data were used in the self-efficacy analysis. Seventy-one participants (87%) submitted their course proposal, and 79 (96%) submitted at least one grant application by the 2-year follow-up. Thirty-three GWC proposals (40%) were funded, and 65 participants (79%) received at least one grant as principal investigator or multiple principal investigator since taking the course. Success rates were equal for men (26 [79%]) and women (34 [79%]) and highest (12 [86%]) for underrepresented faculty. Of the funded proposals, 49 (30%) were NIH R01, R01-equivalent, or R21 awards. Underrepresented participants had the highest (19 [48%]) success rate. Participants’ confidence in the 19 grant-writing skills inventory increased overall. The GWC is a highly effective and innovative program for improving grant-writing success of early-career, women, and underrepresented faculty in academic medicine.

## Teaser

UC San Diego Health Sciences Grant Writing Course provided a structured, step-by-step multi-component experience focused on preparing a competitive grant proposal and improved success rates for men, women, and underrepresented faculty in receiving R01, R01-equivalent, and R21 awards.

The success of early-career faculty at R1 research-intensive institutions depends on the institutions’ ability to establish independent, grant-funded research programs. However, developing the skills necessary to write compelling grant applications is typically not a required part of graduate or medical education. Even when taught pedagogically, there is no substitute for the lived experience of writing persuasive grant applications and the peer review evaluation process. Evaluations of grant applications are highly subjective, and funding decisions are based on many factors.^1^

The competitiveness of the funding environment is also a major factor in grant success. As reported by the National Institutes of Health (NIH), of the 11,463 applications submitted by first-time principal investigators (PIs) in 2023, only 2,174 (19%) were funded by the NIH.^2^ The percentage is similar for previously funded established PIs (4,110 of 20,575 [20%]).^2^ Although receipt of a mentored career development award (K award) has been associated with a greater likelihood of a first independent research grant (R01 or R01-equivalent),^3^ the rates of K-to-R transition during a 10-year follow-up period were recently reported as only 39.9% among faculty in US medical schools and were significantly lower among women compared with men (37.7% vs 41.5%).^4^ Among those receiving their first R01 or R01-equivalent award, the median age at award in 2023 was 45 years for MD and MD-PhD physicians and 41 years for PhD investigators.^5^ Apprehension about one’s ability to succeed in writing fundable grants and having one’s academic salary and career advancement dependent on grant funding are major factors that deter STEM graduates from choosing a career in academic science and medicine.^5-8^ The inequalities in the distribution of NIH funding to women and individuals from historically underrepresented backgrounds are also a concern.^9-12^

To address concerns, many institutions offer various forms of programmatic support to early-career faculty who are preparing their first grant proposal. However, only a few of these programs have evaluated rates of R01 and R01-equivalent grant funding and perceptions of grant-writing self-efficacy. The UC San Diego Health Sciences Office of Faculty Affairs designed and implemented the Grant Writing Course (GWC), a 9-session, intensive course on grant writing, in 2017. The purpose of the GWC is to provide a structured, instructional, step-by-step process focused on preparing a competitive grant application with guidance provided by senior faculty and peer reviewers. Because the program requires institutional resources, it was deemed important to evaluate outcomes from the start. We report here on grant submission rates, funding success, and grant-writing self-efficacy after 2 years of follow-up among 85 early-career faculty who participated in the GWC between 2017 and 2021.

## GWC program description

### Eligibility and recruitment

University of California (UC) San Diego Health Sciences is a research-intensive academic medical institution with 1,879 faculty appointed in the School of Medicine, Skaggs School of Pharmacy and Pharmaceutical Sciences, and Herbert Wertheim School of Public Health and Human Longevity Sciences. Approximately 60% are clinical faculty and 40% are research faculty, 46% are women, and 11% are from underrepresented racial or ethnic backgrounds. Faculty are eligible to participate in the GWC if they have not previously received NIH R01 or R01-equivalent funding as a PI or multiple PI and if they have sufficient research training and a well-defined idea to develop into an R01 or R01-equivalent grant application (Supplementary Material S1). Grant Writing Course is held annually, and recruitment begins with an open call for applications using the Health Sciences faculty email listserv. Application materials are submitted via an online portal and reviewed by the GWC faculty directors. Eligible faculty members with complete applications are accepted into 1 of 3 discipline-specific GWCs: (1) population and behavioral health, (2) basic and translational research, and (3) automation, machine learning, computation, and implementation in health. Class sizes have varied and are ideally 8 to 10 early-career faculty participants.

### Components of the GWC

**Figure 1:**
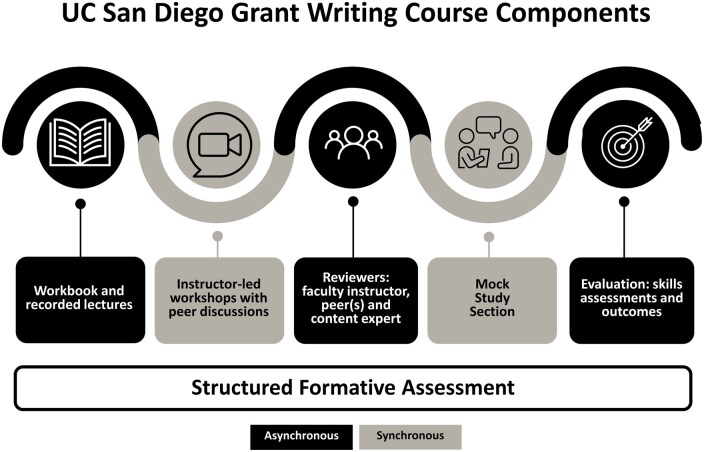
University of California (UC) San Diego Grant Writing Course components. The UC San Diego Grant Writing Course is a 3-month, 9-session, multicomponent course based on structured formative assessment and includes asynchronous (black) and synchronous (gray) components with iterative feedback. Asynchronous activities include reading chapters in *The Grant Application Writer’s Workbook*,^13^ listening to recorded lectures, and peer review. Synchronous interactive components include 8 two-hour faculty instructor-led workshops and a final review of grant proposals in a mock study section. The course is evaluated using preprogram and postprogram skills assessments and grant funding outcomes.

The objectives of the GWC are to develop early-career faculty as competent, skilled grant writers and grant reviewers and to enhance their success rates at obtaining extramural funding. The 3-month course is based on structured formative assessment and guided by *The Grant Application Writer’s Workbook*,^13^ with iterative feedback from senior faculty instructors and content experts 3 times during the course and peer reviewers during each of the eight 2-hour interactive workshop sessions delivered virtually (Figure 1). The GWC curriculum covers all sections of an NIH R01 grant application, including the opening statement, hypothesis, specific aims, significance, innovation, approach sections, preparing other documents (ie, NIH Biosketch), and responding to critiques (Supplementary Material S2). In the mock study section session, senior faculty reviewers provide verbal and written critiques using the current NIH review format, and the last session is focused on responding to critiques. The participants have asynchronous homework supported by the Canvas learning management system, including reading chapters of *The Grant Application Writer’s Workbook*, listening to prerecorded lectures introducing each grant section, submitting written drafts of their own grant, and providing peer review. The course directors of each GWC host relevant senior faculty to lead the sessions, providing unique examples and experiences in preparing and completing those sections. Emphasis is placed on the persuasive, logical, readable, and visually interesting goals of the grant-writing genre. Faculty participants discuss their submitted written sections in peer-review breakout sessions, followed by discussion in the larger class group during which feedback is provided on clarity and structure and questions and concerns are addressed. Participants also gain understanding and experience using NIH web-based resources, contacting NIH program officers for review of draft-specific aims and questions, and learning the grant-review process. The GWC is institutionally funded, with an estimated cost of $118,057 annually, and managed by the Health Sciences Office of Faculty Affairs. Senior faculty (workshop leaders and content experts) receive an honorarium and a service letter for their participation.

### Program evaluation

Evaluation data are collected at 4 timepoints: before beginning the GWC, after taking the course, and 12 and 24 months later. Participants complete a survey that includes a previously developed and validated 19-item Grantsmanship Self-efficacy Inventory that assesses the participant’s confidence in 3 domains: conceptualizing a study, designing a study, and obtaining funding for a study.^14^ Participants responded to the prompt, “We would like to know how confident you are today that you can successfully perform the tasks listed below. Using a 0-10 scale, indicate your level of confidence between No Confidence [0] and Total Confidence [10] in your current abilities in these general areas of grant proposal writing.” This selected instrument has internal consistency and higher levels of self-efficacy that have been associated with an increased probability of submitting a grant within 6 months after training in a grant-writing program.^14^ In the postcompletion evaluation, 10 open-ended questions solicit reflections on the overall value of the course and its various components. At the 12- and 24-month timepoints, participants are asked to report on the number and types of grant proposals submitted and funded since taking the GWC.

### Analysis of grant submission and funding outcomes

Descriptive statistics (numbers and percentages) were used to analyze grant submission and success rates. The data are presented for all GWC respondents who took the course between 2017 and 2021 and by gender and underrepresented status. Responses to the Grantsmanship Self-Efficacy Inventory were analyzed by comparing domain- and item-specific mean scores between the preassessment and the 3 postassessments and calculating the percent change from the preassessment. Reliability coefficients (Cronbach α) were calculated to ensure consistency with the validated measure. Because the evaluation includes the entire population of early-career faculty participants who participated in the GWC between 2017 and 2021 and no sampling or group comparison was involved, we did not use statistical tests. The GWC has continued with cohorts in 2023 through 2025 with a total of 24 participants, but because the 24-month follow-up data are not available for these cohorts the participants were not included in the analysis.

### Analysis of open-ended feedback

At the conclusion of the GWC, 55 of the participants provided 455 open responses to 10 questions across all years. The questions asked participants to “reflect on” course components (ie, content, instructors, pacing), in-class critique or peer review and mock study sessions, and improvements needed. To identify meaningful patterns and generate knowledge about the data, thematic analysis was conducted using ATLAS.ti, version 25 (ATLAS.ti Scientific Software Development GmbH)^15^ and coded into 8 themes. Qualitative data analysis in the form of a thematic analysis involves identification of themes and patterns that appear in otherwise unstructured qualitative data.^16^ Qualitative analysis was conducted independently, led by the Office of Faculty Affairs director for research and evaluation and a senior staff research associate, maximizing rigor to the approach. The 10 questions were compiled into 4 domains: (1) feedback sessions (ie, peer review, in-class group critique, content expert), (2) content and structure (ie, course content, pacing, asynchronous or synchronous learning), (3) perceptions of faculty instructors and leadership (ie, quality of lectures, diversity), and (4) overall comments and recommendations. ATLAS.ti was used to develop and narrow codes. An iterative process to review and analyze the codes resulted in 8 emergent themes representing the data.

## Summary of evaluation data

### Participants

A total of 85 early-career faculty participated in the GWC between 2017 and 2021, 45 women (53%) and 34 men (40%) with 15 (18%) self-identifying as being from historically underrepresented racial or ethnic groups (Supplementary Material S3). Of the 85 faculty participants, 24 had MDs (28%), 24 had MDs with a master’s degree and/or PhD (28%), and 37 had PhDs (44%). Most of the participants were assistant professors within 2 to 3 years of their initial faculty appointment from the UC San Diego School of Medicine, Skaggs School of Pharmacy and Pharmaceutical Sciences, and Herbert Wertheim School of Public Health and Human Longevity Science. The participants included individuals with a wide range of experience; some may have received an NIH K award and had experience writing an NIH R-type award, and others may have had limited experience in grant writing.

### Grant submission and funding success

Follow-up surveys were completed by 82 participants (98%) at 12 months and 77 (91%) at 24 months, and data were in the analysis of grant-funding outcomes. By the 2-year follow-up, 71 participants (87%) had submitted the proposal worked on during the GWC, and 79 participants (96%) had submitted at least 1 grant application (Table 1). A total of 33 (40%) of the GWC-specific grants had been funded, and 8 (10%) were discussed but not funded. A total of 65 participants (79%) had received at least 1 grant as a PI or multiple PI since taking the GWC. Grant submission rates were very high among women (42 [98%]) and underrepresented (13 [93%]) faculty. Grant funding rates were similar for women (34 [79%]) and men (26 [79%]) and higher among underrepresented faculty (12 [86%]) than nonunderrepresented faculty (52 [78%]). A total of 460 grants had been submitted overall, and 163 (35%) of these had been funded (Table 2). Of the 163 funded grants reported, 49 (30%) were NIH R01, R01-equivalent, or R21 awards, 40 (25%) were foundation or industry grants, 38 (23%) were internal UC San Diego grants, and 14 (9%) were NIH K-type or other career development awards. Underrepresented faculty received the highest (19 [48%]) number of R01, R01-equivalent, or R21 grant awards.

**Table 1:** Outcomes at 12 or 24 months among 82 University of California San Diego Health GWC participants enrolled from 2017 to 2021.^a^

Outcome	**All GWC respondents** ** (N = 82)**	**Women ** **(n = 43)**	**Men** ** (n = 33)**	**UR** ** (n = 14)**	**Non-UR** **(n = 67)**
**Participant outcomes of GWC grant proposal, no. (%)**					
** Funded**	33 (40)	17 (40)	14 (43)	6 (43)	26 (39)
** Discussed but not funded**	8 (10)	3 (7)	5 (15)	0	8 (12)
** Not discussed**	23 (28)	12 (28)	8 (24)	6 (43)	17 (25)
**Submitted or resubmitted-pending review**	7 (9)	4 (9)	3 (9)	1 (7)	6 (9)
** Not submitted**	11 (13)	7 (16)	3 (9)	1 (7)	10 (15)
**Participant outcomes for all grants submitted since GWC, no. (%)^b^**					
**Submission of at least 1 grant (as PI or MPI)**	79 (96)	42 (98)	31 (94)	13 (93)	65 (97)
** At least 1 grant funded (as PI or MPI)**	65 (79)	34 (79)	26 (79)	12 (86)	52 (78)
** No grant funded (as PI or MPI)**	17 (21)	9 (21)	7 (21)	2 (14)	15 (22)

Abbreviations: GWC, Grant Writing Course; MPI, multiple principal investigator; PI, principal investigator; UR, underrepresented.

a Of the total 85 GWC participants, 82 respondents completed the 12-month survey and 77 completed the 24-month survey. Six respondents chose not to disclose gender information, and 1 respondent chose not to disclose underrepresentation.

b All grants are inclusive of GWC proposals and other grants.

**Table 2:** Grants submitted and funded among 82 University of California (UC) San Diego Health GWC participants enrolled from 2017 to 2021.^a^

Grants	No. (%) of grants				
	**All GWC respondents ** **(N = 82)**	**Women** ** (n = 43)**	**Men** ** (n = 33)**	**UR** ** (n = 14)**	**Non-UR** ** (n = 67)**
**Total no. of grants submitted since GWC completion**	460	260	173	84	370
**Total no. of grants funded since GWC completion**	163	91	63	40	119
**Grants funded by category, no. (%)**					
**NIH R01, R01-equivalent,^b^ or R21 awards**	49 (30)	29 (32)	19 (30)	19 (48)	30 (25)
**Foundation or industry grants**	40 (25)	19 (21)	19 (30)	8 (20)	32 (27)
**UC San Diego grants (ie, Academic Senate, ACTRI)**	38 (23)	27 (30)	10 (16)	7 (18)	31 (26)
**NIH K-type or other career development grant**	14 (9)	6 (6)	7 (11)	3 (7)	11 (9)
**Unknown**	22 (13)	10 (11)	8 (13)	3 (7)	15 (13)

Abbreviations: ACTRI, Altman Clinical Translational Research Institute at UC San Diego; GWC, Grant Writing Course; NIH, National Institutes of Health; UR, underrepresented.

a Of the total 85 GWC participants, 82 respondents completed the 12-month survey and 77 completed the 24-month survey. Six respondents chose not to disclose gender information, and 1 respondent chose not to disclose underrepresentation.

b R01-equivalent grants include DP1, DP2, DP5, R01, R37, R56, RF1, RL1, U01, and R35 activity codes.

### Self-efficacy and satisfaction

The Grantsmanship Self-Efficacy Inventory was completed by 75 GWC participants (88%) at all 4 timepoints (Table 3). Consistent with the original measure, the inventory has high internal consistency among GWC participants (overall α = .95, conceptualizing α = .92, designing α = .94, funding α = .86). Overall, participants’ confidence increased for all 19 grant-writing skills. Improvements were greatest immediately after the course and remained robust, although with modest decreases at the 12- and 24-month follow-ups. The highest percentage increase in confidence was for describing a major funding agency’s proposal review and award process (76% improvement).

**Table 3:** Grantsmanship confidence self-efficacy inventory at pre-post University of California San Diego Grant writing course completion, 12- and 24-month follow-up.

How confident are you today that you have the skills to…	Prescore (total confidence)	Postscore, % increase	12-mo postscore, % increase	24-mo postscore, % increase
**Funding a study**				
**Describe a major funding agency’s (eg, NIH, foundation) proposal review and award process**	4.83	76	63	63
**Speak with a person at the funding agency regarding your project or project ideas**	4.96	64	50	52
**Identify appropriate funding sources (local, state, national) to support a study**	5.25	54	44	44
**Write a competitive grant application**	5.32	51	45	38
**Total**	5.09	61	50	49
**Designing a study**				
**Determine the universe, population, and appropriate sample for a given study**	5.75	39	31	33
**Determine an adequate number of participants for your research project**	5.47	40	35	33
**Design the best data analysis strategy for your study**	5.81	36	32	29
**State the purpose, strengths, and limitations of each study design**	6.28	31	26	27
**Choose an appropriate research design that will answer a set of research questions and/or test a set of hypotheses**	6.15	32	25	26
**Select methods of data collection appropriate to the study population and variable(s) of interest**	6.27	29	28	26
**Determine how each variable will be measured**	6.52	27	22	21
**Total**	6.04	33	28	28
**Conceptualizing a study**				
**Convince grant reviewers your proposed study is worth funding**	4.71	62	53	46
**Relate specific questions of interest to underlying theory**	6.28	32	28	28
**Organize your proposed research ideas in writing**	6.27	34	26	27
**Place your study in the context of existing research and justify how it contributes to important questions in the area**	6.51	30	26	26
**Articulate a clear purpose for the research**	6.51	31	25	24
**Refine a problem so it can be investigated**	6.72	22	18	19
**Develop a logical rationale for a particular research idea**	6.92	21	15	16
**Select a suitable topic area for study**	7.27	15	12	12
** Total**	6.40	29	24	24

Abbreviation: NIH, National Institutes of Health.

Thematic analysis of open-text responses from 55 participants resulted in the emergence of 8 high-level themes across the 4 analytic domains (Table 4). Notably, participants’ open-text responses were largely favorable, endorsing the value of participating in the GWC and complementing quantitative findings regarding grant-writing success and self-efficacy. Participants highly recommend the course and highlight its impact on their subsequent successes. The course was described as a “wonderful experience,” “helpful,” “instrumental in securing funding,” and “fantastic.” The mock study section was highlighted as a valuable and unique opportunity. Suggestions for improvements included participants arriving with a well-developed grant idea or draft to keep pace with the program, devoted time or declining nonessential commitments during the course, better structured reviewer feedback, and a grants repository. See Table 4 for themes, definitions, and supportive quotations.

**Table 4:** Analysis of open-ended feedback and short answer themes among 55 UC San Diego Health GWC participants enrolled from 2017 to 2021.

Theme	Description	Quotation
**High recommendation and impact**	Participants overwhelmingly recommend the GWC, noting significant improvements in their grant-writing skills and increased confidence. Many reported successful funding outcomes due to their program participation, such as securing multiple R01 grants.	“The GWC was hands-down fantastic! It was a tremendous help for me to 1) discuss my grant proposal with my peers and hear their feedback and inspect their perspective, and to 2) witness the mock study section and realize how a grant actually moves from writing to submission, to discussion, to presentation in the study section meeting. This greatly helped my grant writing and my funding success. I submitted the grant that got awarded after GWC (NIH DP2) before GWC (not funded) and then a drastically revised version after GWC (funded! yay!). So I cannot thank GWC enough.”
**Course structure and content**	Participants suggested that the course is well-organized and comprehensive, with a special emphasis on the mock study sessions.	“The content and structure of the course was great. I went from having no idea about how to navigate the NIH grant application process to writing and now about to submit my first NIH grant application—thank you!”
**Preparation and timing**	Participants suggested arriving with a well-developed grant idea or draft to keep pace with the program. Noted areas for improvement included extending timelines for assignments and overall course duration would allow for better preparation and absorption of materials.	“The GWC was an excellent course and I really appreciate the time and effort put in by the faculty to make this happen…The only feedback I would have is that the timelines for each stage were too short, and this course should extend over a longer period of time to allow adequate time to prepare the sections.”
**Quality of feedback**	Feedback from peers and faculty was crucial for developing strong grant proposals. However, participants expressed a desire for more structured feedback sessions and clearer expectations for peer reviews.	“The peer review process was useful but would have preferred working with a new peer every 2-3 sessions as opposed to every class.”
**Diversity of ­instructors**	The diverse backgrounds and expertise of the instructors were highlighted as strengths of the experience, enriching the learning process through multiple diverse perspectives.	“I thought the quality was overall very good, and I did value the diversity of instructors. It made the class very engaging and dynamic.”
**Challenges faced**	Many participants struggled to balance course demands with clinical responsibilities, especially during the pandemic. The online format was sometimes perceived as less engaging.	“The virtual aspect of this course left a bit to be desired. Nothing that the course could do about this with Covid. But it did make it a bit less engaging.”
**Confidence building**	Participants reported a notable increase in confidence regarding grant writing and navigating the funding process, even if they did not secure funding immediately.	“Probably I will feel more confident once I actually get a grant funded, but I believe that the GWC significantly improved my confidence in most of these areas.”
**Suggestions for improvement**	Recommendations included extending the course duration, providing a repository of successful grant examples, and offering more tailored support and feedback on grants.	“Having reviewers critique a grant that I had not read wasn’t particularly helpful. One-on-one time would be more helpful.”

Abbreviations: GWC, Grant Writing Course; NIH, National Institutes of Health.

## GWC outcomes in context

Although grant-writing programs have increased at US academic institutions, published systematic evaluations are still few and most rarely consider gender and race or ethnicity. At the University of Minnesota Medical School, the well-established Proposal Preparation Program (P3) has several components in common with the GWC. Of 194 past participants as of 2018, 88% had submitted their P3 NIH grant (R- and K-type), with 35% reporting funding success.^17^ These outcomes are similar to those of the UC San Diego GWC, but the follow-up time after P3 completion was not reported. At Duke University, an evaluation of a 20-hour curriculum with similar GWC components found that grant writing success rates were 28% for submitted R applications and 64% for submitted K awards, a large increase over previous success rates.^18^ Overall, these programs have been evaluated as successful when outcomes exceed the usual 17% to 19% R01 success rate for first-time PIs in the last 5 years^2^ as we observed with the GWC.

A large analysis of 6 grant-coaching programs within the NIH’s National Research Mentoring Network^19^ that were intentionally designed to improve the demographics in NIH grantees reported outcomes for 2015 to 2019 for 545 investigators across 187 institutions.^20^ In this group, 59% of participants submitted at least one grant application and 41% received funding. The funding rate for the 74 NIH grants submitted at the time of the report was 22% (n = 16). Although the GWC demonstrates success of all early-career faculty in obtaining NIH funding, underrepresented participants received the highest (19 [48%]) success rate in NIH R01, R01-equivalent, or R21 awards.

### MD vs PhD

Type of terminal degree is a source of heterogeneity in grant-writing success. Individuals with MDs on average receive their first NIH award several years later than those with PhDs,^5^ which is likely due to extended clinical training, lack of protected time to perform research among academic physicians with clinical roles, and limited preaward administrative and budgetary assistance. Training through institutional clinical and translational research institutes is one form of institutional support, and some offer grant-writing programs focused mainly on K awards^21^ or R awards.^22^ The GWC requires research training or at least some experience conducting research for clinicians to participate. The course is not a substitute for research training. For physicians without research training who are expected or want to write grants as PIs, a path to grant writing that includes research training through a master’s program or other means will be critical to success for most faculty. It is noteworthy that the GWC included a rate of 56% for those with MDs or MDs with a master’s or PhD, which shows that the program has supported physician-scientists pursuing a clinician research career path.

### Environment

The research capacity and intensiveness of the institution in which grant-writing programs are offered are also likely factors in the grant-writing success rates of participants. At UC San Francisco, for example, a longstanding presubmission grant review program had a 44% success rate during a 12-month period.^23^ This program does not include a structured application-development component, so the faculty must have the capability of bringing a finished grant to review 2 to 4 weeks before the grant deadline, which likely selects for more capable and experienced faculty. In contrast, an evaluation of pilot programs, including grant-writing workshops of various designs offered at 9 low-resourced (<$7.5 million total NIH research funding) institutions supported by NIH’s Building Infrastructure Leading to Diversity (BUILD) program, found no evidence of an increased number of submitted or funded grants of early-career faculty analyzed at the institution level, although the numbers of presentations and publications had increased.^24^ The GWC program depends on close engagement with research content experts and experienced, well-funded senior faculty to guide faculty participants in navigating the twists and turns of the grant-writing process to success. Having a critical mass of grant-funded faculty members is thus a key factor in replication of the program in other institutions.

### Reflections

Although the benefits of extramural funding levels are important to institutions, it is also critically important to acknowledge the many other gains of implementing the GWC program, such as improving faculty morale, community, and, importantly, equity in grant application success. The GWC program increases awareness of research-active faculty across health sciences and thus promotes collaboration. The program increases opportunities for senior faculty to pass on effective strategies and experiences gained over decades, including faculty outside one’s usual orbit or sphere of expertise. In fact, many senior faculty report that their involvement in the course and exposure to other senior faculty’s methods improve their own grant-writing skills. Thus, the program supports the development of a supportive environment for grant-writing success, which gets away from the solitary, unsupported experience typical for early grant writers, enabling the success of all faculty, including women and underrepresented participants. As stated by Dr Beronda L. Montgomery in her book *Lessons From Plants*,^25^ “Supporters, mentors and leaders matter greatly in helping individuals reach their full potential.…Given two individuals of equal aptitude, the one connected to the right resources or embedded in an appropriate developmental or support network is much more likely to success.” In our view, these intangible returns on investment are priceless.

## Strengths and limitations

Strengths of this evaluation include the 2-year prospective follow-up of GWC participants with a high response rate and minimal missing data. We include both a quantitative assessment of grants submitted and awarded and self-evaluations of grant-writing self-efficacy and qualitative analysis of participant feedback. Our focus is on the value of the GWC for supporting the grant-writing success and career development of our early-career faculty. We have not attempted to compare the 79% success rate in receiving a grant award with rates in a comparable control group. The GWC is a multicomponent program, and we cannot separate out the effects of any one component to this success. These limitations can be overcome by using experimental designs with randomization of faculty participants to different grant-coaching groups to unpack the most effective parts of a grant-coaching program.^26^ We also have not attempted to quantify the financial return on investment in this report, which would require data on the dollars awarded to GWC participants vs a matched comparison group of some type, quantification of the investment of time and effort of staff and faculty, and a proper cost-effectiveness analysis, all beyond the scope of our data and this analysis.

## Conclusions

We conclude that the GWC was highly successful in supporting early-career faculty to submit grants, compete successfully for research funding, and improve their grant-writing self-efficacy. The fact that success rates were similarly high in men and women and particularly high in underrepresented faculty is a particular strength of the GWC given the documented disparity in women and underrepresented investigators receiving NIH funding.^9-12^ Institutional support for programs of this type is an important component of promoting the equitable success of early-career faculty in garnering extramural funding.

## Supplementary Material

wvaf031_Supplementary_Data

## Data Availability

The authors confirm that the data supporting the findings of this study are available within the article.

## Previous presentations

None declared.
