# Supplementary material for: Building strong grant writers in academic medicine: outcomes of early-career faculty enrolled in the University of California San Diego Health Sciences Grant Writing Course
Source: Acad Med. 2026 Jan 12;101(1):34–42. doi: 10.1093/acamed/wvaf031 (PMC12851622; doi:10.1093/acamed/wvaf031)
Supplement: wvaf031_Supplementary_Data [file wvaf031_supplementary_data.pdf]

Supplementary Material 1

UC San Diego Health Sciences Grant Writing Course (GWC) | Application

Participation in GWC requires attending and actively participating in 9 online sessions over a 3-month period as well as completing asynchronous work and several hours per week of homework.

The program is an **intensive**, instructor-led course based on structured, formative assessments using "*The Grant Application Writer's Workbook, NIH version*." The course is specific to **NIH R-type or K-type** grants (or equivalent), and you must be actively working on a research proposal with some preliminary data.

As a GWC participant, you will complete weekly homework assignments in preparation for weekly group Zoom sessions, participate in peer-to-peer group discussions, instructor led discussions and meet with a content expert for guidance – selected by the participant. Your final grant proposal draft will be reviewed by a mock study section comprised of NIH-funded senior professors in Health Sciences.

Priority will be given to Health Sciences faculty who are/have:

- Salaried appointment >50%
- Writing R or K mechanism, or equivalent (i.e., foundation grant)
- Assistant and Associate Professors
- Never been awarded an R01 or R01-equivalent grant as PI or MPI
- Faculty with research training and actively engaged in research
- Faculty who can identify a content expert or collaborator team who can assist with their grant development
- Faculty who can identify a mock section study reviewer to evaluate their proposal

Please note that a draft of the **Specific Aims** page is required as part of the GWC application.

Because we have limited capacity and this course is only offered yearly, we will contact applicants regarding next steps after all applications have been reviewed.

We look forward to enhancing your skills in NIH grantsmanship and helping to increase the acceptance rate of your grant submissions.

-Office of Faculty Affairs
hsfacultyaffairs.ucsd.edu | gwc.ucsd.edu

Name and Email

Please upload your **Specific Aims** (1 page), which includes the **title and hypothesis**, and **Preliminary Data** (1 page). **Please include your full name in the file name.**

Please upload your **current CV**. **Please include your full name in the file name.**

Have you successfully competed for an NIH R01 or R01-equivalent award as a PI or MPI? R01-equivalent include DP1, DP2, DP5, R01, R37, R56, RF1, RL1, U01 and R35 activity codes

If yes, please explain why you would still like to be considered for this course.

Content Expert
Admitted participants are required to recruit a **Content Expert** (UC San Diego or external faculty researcher), who can meet with them to discuss ideas and to review the grant components throughout the course. The Content Expert is also required to submit a **written NIH critique** of the participant's grant proposal to the program prior to the Mock Study Section meeting. Please provide the name, institution, and department of an individual you'd like to recruit as your Content Expert should you be admitted to take the course.

Research Mentor #1
Please list the contact information of your current research mentor at UC San Diego, if you have one. We may reach out to your mentor regarding their interest in serving as a senior faculty instructor.

Research Mentor #2
Please list the contact information of your second research mentor at UC San Diego, if available. We may reach out to your mentor regarding their interest in serving as a senior faculty instructor.

Mock Study Section Reviewer
The GWC hosts a mock study section for its participants as a required component of the course, and we ask the participants to identify at least 1 UC San Diego faculty member to serve as a reviewer of their grant. Please identify and list contact information for faculty who can potentially serve as your GWC mock study section reviewer, should you be admitted to the course. This individual should have a good track record of grant funding and content expertise in your area of study.

Please describe your research infrastructure (access to datasets, lab, and personnel).

Please indicate your percent time dedicated to research and clinical activities.

Choose the area of study which best fits your GWC proposal.

- ○ GWC1 Population and Behavioral Health
- ○ GWC2 Basic and Translational Research
- ○ GWC3 Machine-learning, Automation, Computation, and Implementation Science

What grant mechanism would you be interested on working on?

- ☐ K01
- ☐ K08
- ☐ K23
- ☐ R01
- ☐ R03
- ☐ R21
- ☐ R35
- ☐ Other
- ☐ Unsure

Please note once you click the "SUBMIT" button, you will no longer be able to edit your application.

Supplementary Material 2

UC San Diego Health Sciences Grant Writing Course (GWC) | Components

Course Book: The Grant Application Writer's Workbook (NIH Version)
**Recorded lectures and assignments available on Canvas platform*

	Content	Homework
**Pre-Course**	N/A	• Read Chapters 1-4 of the Grant Application Writer's Workbook<br>• Complete pre-assessment<br>• Email content expert mentor contact information to the course program manager
**Class 1**	Opening Statement and Hypothesis	• Read Chapter 6 and view Canvas recorded lecture 1<br>• Submit the target funding mechanism and planned submission date<br>• Submit draft of Opening Statement, Hypothesis, and Specific Aims<br>• Review your partner's draft<br>• Prepare a 5-minute PowerPoint presentation (3-5 slides) introducing your research, record on Zoom, and upload on Canvas
	• Instructor feedback on Opening Statement, Hypothesis, and Specific Aims provided	
**Class 2**	Specific Aims	• Read Chapters 7-8 and view Canvas recorded lecture 2<br>• Submit revised Opening Statement, Hypothesis, and Specific Aims<br>• Review your partner's revised draft<br>• Comment on assigned peers' PowerPoint presentation
**Class 3**	Significance & Innovation	• Read Chapters 9-10 and view Canvas recorded lecture 3<br>• Submit draft of Significance & Innovation and revised Opening Statement, Hypothesis, and Specific Aims<br>• Review your partner's Significance & Innovation section
	• Instructor feedback on Significance & Innovation provided	
**Class 4**	Approach 1	• Read Chapter 11 and view Canvas recorded lecture 4<br>• Submit draft of Approach and revised Specific Aims and Significance & Innovation<br>• Review your partner's Approach section
**Class 5**	Approach 2	• Submit revised Specific Aims, Significance & Innovation, and Approach<br>• Review your partner's revised Approach section
	• Instructor feedback on Approach provided	
**Class 6**	Title, Narrative, Abstract, Summary	• Read Chapters 18-19 and view Canvas lecture 5<br>• Submit draft of Title, Narrative, Abstract, and Summary and all revised prior sections.<br>• Review your partner's Title, Narrative, Abstract, and Summary
**Class 7**	Other Documents	• Read Chapters 12-13, 15-17, 20 and view Canvas recorded lecture 6<br>• Submit draft of Other Documents (BioSketch, Budget, Facilities, Equipment, Human Subjects, Animals, Resource Sharing Plan, Authentication, Cover Letter) and all prior revised sections<br>• Review your partner's Other Documents
**Class 8**	Mock Study Section	• Submit a single PDF of the final draft of your grant proposal, NIH Biosketch, Vertebrate Animals (if applicable) to be routed to your reviewers<br>• Submit your content expert's grant critique (use NIH critique template)
**Class 9**	Responding to Critiques	• View Canvas recorded lecture 7<br>• Submit Introduction to the revised application (1 page)

Supplementary Material 3

Demographics of Grant Writing Course Participants enrolled from 2017-2021 (n=85)

Characteristic	No. of Participants (%)
**Gender**	
Women	45 (53%)
Men	34 (40%)
Decline to state	4 (5%)
Unknown	2 (2%)
**Under-represented**	
Under-represented	15 (18%)
Not underrepresented	69 (81%)
Unknown	1 (1%)
**Degrees**	
MD	24 (28%)
MD + master's or PhD	24 (28%)
PhD	37 (44%)
